# A collaborative effort of China in combating COVID-19

**DOI:** 10.1186/s41256-020-00174-z

**Published:** 2020-11-11

**Authors:** Mohamed S. Bangura, Maria J. Gonzalez, Nasra M. Ali, Ran Ren, Youlin Qiao

**Affiliations:** 1 School of Public Health, Dalian Medical University, Dalian, 116044 China; 2 Dalian Maternal and Child Health Hospital, Dalian, 116033 China; 3 Global Health Research Center, Dalian Medical University, Dalian, 116044 China; 4 Department of Cancer Epidemiology, National Cancer Center /Cancer Hospital, Chinese Academy of Medical Sciences and Peking Union Medical College, Beijing, 10002 China

**Keywords:** Collaborative effort, COVID-19, Multisectoral, China

## Abstract

A novel coronavirus (COVID-19) was firstly identified in Wuhan by the end of 2019. China has implemented a series of preventive measures to deter COVID-19 spread and its consequences since the beginning of the epidemic. In China, the expansion of COVID-19 has been slowed down significantly through the effort of all contributors, including governments, nongovernments, and civil society. All collaborators have been actively involved in combating the epidemic, using their respective strengths to play their roles. China has mitigated the number of cases due to the mobilization of the whole society and collaborators joining collective efforts and actions in solidarity to tackle and conquer the virus. To date, China has continued to implement actions to control any resurgence of new cases of COVID-19 and keep its population safe. The people’s united effort against the virus has enhanced a great insight into China, and it might serve as a model to the global community in fighting the COVID-19 pandemic  in terms of coordination, solidarity, decisiveness, and leadership.

## Background

The end of 2019 stunned China with an emerging health outbreak that appalled the Chinese population and the global community. The coronavirus (COVID-19) occurrence in Wuhan, was declared a Public Health Emergency of International concern and it resulted in a rapid rate of transmission [1]. Therefore, the government issued and implemented policies, such as unique mechanisms to track patients with the virus.

Since early April, China has mitigated the expansion of COVID-19 inside the country. Central and local governments took actions to institute several preventive measures to deter COVID-19 spread and consequences [2]. The citizens also played a role by following the regulations and being committed to the measures implemented. Thus, the control of the outbreak has been achieved through the effort of all collaborators. As the virus spread globally in a short time, changing into a pandemic emergency, it raised concern over how developing countries would take action to control the pandemic. This commentary aims to demonstrate China’s collaborative efforts in controlling the spread of COVID-19 and share practices that can be helpful for the global community.

## Actions of government authorities

As a first measure, the government locked down Wuhan and encouraged people to shut down their businesses and daily activities, staying indoors to contain the spread. Local travels and flights were banned. When the number of cases started to increase in China, other cities also closed access from other residents entering to keep their citizens safe. Social activities and meetings decreased since social distancing measures were implemented in the whole country, including canceling sporting activities and closing up theaters, bars, restaurants, malls, among other places. In addition, such a massive lockdown system in other cities resulted in putting considerable millions of people in a nation-wide quarantine and yielded a beneficial outcome towards combating against the virus [3]. To address the challenge of a shortage of hospital beds for infected patients, the government rapidly implemented a policy for building temporary hospitals in Wuhan. Two notable hospitals, the 1000-bed Huo Shen Shan Hospital and the 1600-bed Lei Shen Shan Hospital, were built within ten days [3].

## Actions of schools and students

Academic institutions took preventive actions in extending the winter vacation for all students. Those living on campus, have been restricted from going out, and the universities provide them with food, masks, and other necessary supplies. Moreover, the students adhere to control measures and support one another to manage their lives. In the beginning of the pandemic, leaders were formed to monitor compliance in the dormitories, such as checking the body temperature of every student. Most importantly, psychological counseling service has been available in most universities to deal with the panic of the outbreak. As of August 29th, most schools and universities remain closed, and many of them have implemented online classes and live-stream teaching to continue education from a distance.

## The dedication and actions of healthcare workers

Healthcare workers have been fighting on the front line to provide treatment for infected people, while providing residents with anti-epidemic preventive care, disinfection, publicity, and education. Around 42,600 healthcare professionals from all over China were sent to the center of the outbreak in Wuhan to collaborate with the local healthcare workers in combating the epidemic [4]. In addition, the outbreak demanded a rapid and effective response, which led to the intensive and immediate training of doctors, nurses, and other medical staff from different specialties for combating the disease. Furthermore, the medics treating infected patients in isolation wards and the intensive care unit in Wuhan were available 24 h a day, and many of them abandoned their personal activities and families to answer the call of this national service [5]. Moreover, as the pandemic keeps ravaging other countries, Chinese medical experts continue being committed to controlling the disease, and they are sharing their experiences with other front-liners abroad, either via video platforms or traveling overseas to directly assist in medical care.

## Actions of enterprises

A corporate response to the outbreak focused on supply chain logistics because it is essential in a crisis to ensure the availability of enough supply of materials, including surgical masks, disinfectants, and protective suits for the front-line medical workers. Corporations, including Alibaba, Bank of China, ByteDance, and others donated volumes of healthcare gears and other supplies to the affected areas. Manufacturer companies set up makeshift assembly lines to produce additional medical supplies [6]. In addition, Huawei and China Telecom set up a 5G-enabled remote video diagnostic center, enabling medics to conduct remote online consultations with potential patients. Companies like Alibaba, Tencent, Baidu, ZTE, JD.com, and iFLYEK all implemented technologies as big data in medical services [6]. Furthermore, Information, Communications, and Technology (ICT) have been employed to tackle the epidemic, such as online epidemic information dissemination platforms, Artificial Intelligence (AI), temperature monitoring, and consultation platforms, AI-assisted radiological image interpretation, and intervention recommendations, big data analytics for epidemic prevention and control, supercomputing for vaccine and drug development, and 5G-based robotics and infrastructure [7].

## Actions of scientists and scholars

The Scientific Community in China, in collaboration with other countries, published and analyzed genomes of the causative agent of COVID-19 and concluded that it might have originated from bats [8]. China has published several clinical guidance manuals and prevention Handbooks in Chinese and English, to collaborate with the global governance of the epidemic. The scientific research team of Shanghai Jiaotong University has provided strong technical support for the release of the first new coronavirus detection kit in China through the application of nanometer magnetic carriers developed over several years [9]. Moreover, the Chinese and international scientists are working rapidly on developing a vaccine against COVID-19 [10].

## Actions of the community

As the most grass-roots organization in government, community works monitored residents for signs of illness, screening them for treatment and quarantine, providing food and daily necessities, and bolstering residential checkpoints to guard outsiders who might bring in the virus. The crucial action for citizens was the improvement in personal protection, encouraging people to cover their mouth and nose while coughing or sneezing, avoiding public spitting, regular hand hygiene with water and soap or alcohol-based sanitizer, and wearing face masks to go out, which was mandatory for all inhabitants. Moreover, numerous volunteers assisted in providing enhanced communications and protective strategies for further containment. Actionable information and detailed procedures were shared at the entrance of communities to ensure the health of the population, especially vulnerable people most at risk such as elders. Some of these actionable information and detailed procedures included self-health improvement, self-protection, disease identification, and guidance for help-seeking if needed. These containment measures were only effective due to the collective compliance of the citizens.

## Actions implemented after revoking the lockdown

Some new measures have been implemented; residents have to show an application code that will indicate their virus-free status before entering public buildings and hospitals that will disclose the health condition and whereabouts for the last 14 days. At the end of August, 2020 schools and universities across the country started to reopen with caution and strict preventive measures, including disinfectant chambers, body temperature checking, sanitizing daily utilities, and a proper social distancing in the classrooms. Many people have returned to work, and most of the provinces have asked their workers to perform a nucleic acid test to ensure their virus-free condition. On the other hand, to avoid imported cases, Chinese provinces and cities implemented different quarantine policies, such as quarantining travelers from overseas in designated hotels. In addition, since the end of March, the entry of foreign nationals has been temporarily suspended.

## Implications of the actions taken by all levels in China

China has slowed down and controlled the spreading of the virus inside the country due to the mobilization of the whole society joining collective efforts and actions in solidarity to conquer the virus. It is essential to point out that the Chinese whole-of-government approach and social mobilization system are the necessary guarantees for controlling the pandemic. The sense of a community with a shared goal reinforces the joint effort against the epidemic in China. It is with a strong conviction that the people’s united effort against the virus has enhanced a great insight into China, and it might serve as a model to the global community.

## Data Availability

Not applicable.

## Abbreviations

COVID-19: Coronavirus Disease 2019
ICT: Information Communication Technologies
AI: Artificial Intelligence
